# Sepsis modulates cortical excitability and alters the local and systemic hemodynamic response to seizures

**DOI:** 10.1038/s41598-022-15426-w

**Published:** 2022-07-05

**Authors:** Lorenzo Ferlini, Antoine Nonclercq, Fuhong Su, Jacques Creteur, Fabio Silvio Taccone, Nicolas Gaspard

**Affiliations:** 1 Department of Neurology, Erasme Hospital, Université Libre de Bruxelles, Route de Lennik, 808, 1070 Brussels, Belgium; 2 Bio-, Electro- And Mechanical Systems (BEAMS), Université Libre de Bruxelles, Avenue F.D. Roosevelt 50 CP165/56, 1050 Brussels, Belgium; 3 Department of Intensive Care, Erasme Hospital, Université Libre de Bruxelles, Route de Lennik, 808, 1070 Brussels, Belgium

**Keywords:** Neuroscience, Neuro-vascular interactions

## Abstract

Non-convulsive seizures and status epilepticus are frequent and associated with increased mortality in septic patients. However, the mechanism through which seizures impact outcome in these patients is unclear. As previous studies yielded an alteration of neurovascular coupling (NVC) during sepsis, we hypothesized that non-convulsive seizures, might further impair NVC, leading to brain tissue hypoxia. We used a previously developed ovine model of sepsis. Animals were allocated to sham procedure or sepsis; septic animals were studied either during the hyperdynamic phase (sepsis group) or after septic shock occurrence (septic shock group). After allocation, seizures were induced by cortical application of penicillin. We recorded a greater seizure-induced increase in the EEG gamma power in the sepsis group than in sham. Using a neural mass model, we also found that the theoretical activity of the modeled inhibitory interneurons, thought to be important to reproduce gamma oscillations, were relatively greater in the sepsis group. However, the NVC was impaired in sepsis animals, despite a normal brain tissue oxygenation. In septic shock animals, it was not possible to induce seizures. Cortical activity declined in case of septic shock, but it did not differ between sham or sepsis animals. As the alteration in NVC preceded cortical activity reduction, we suggest that, during sepsis progression, the NVC inefficiency could be partially responsible for the alteration of brain function, which might prevent seizure occurrence during septic shock. Moreover, we showed that cardiac output decreased during seizures in sepsis animals instead of increasing as in shams. The alteration of the seizure-induced systemic hemodynamic variations in sepsis might further affect cerebrovascular response to neuronal activation. Our findings support the hypothesis that anomalies in the cerebral blood flow regulation may contribute to the sepsis-associated encephalopathy and that seizures might be dangerous in such a vulnerable setting.

## Introduction

Critically ill patients with sepsis may sometimes experience non-convulsive seizure (NCSz) and non-convulsive status epilepticus (NCSE)^1,2^. Some studies also reported an association between the occurrence of seizure or periodic epileptiform discharges and increased mortality^1,2^ in patients with sepsis; however, the mechanisms through which NCSz and NCSE might impact outcome in this setting have not been elucidated. Also, infections and sepsis are frequent and associated with worse outcome in patients admitted to an intensive care unit (ICU) with NCSE^3^, suggesting a significant interplay between the infectious and epileptic events on mortality.

In brain-injured patients, NCSz are associated with cerebral hypoxia and metabolic stress, in part due to alteration in neurovascular coupling (NVC)^4,5^. NVC refers to the compensatory mechanisms that adjusts cerebral blood flow (CBF) to neuronal activity and metabolic demand to different stimuli^6^. Evidence in the literature suggests that ictal activity is coupled with a prominent increase in CBF^7,8^, although NVC seems to be transiently unable to fully support cortical activation at seizure onset and a transient dip in tissue oxygenation has been described^7^. Human and animal studies have found an impairment of NVC during sepsis or septic shock, both for spontaneous^9^ or stimulus-induced cortical neuronal activation^6^. As NCSz consist of increased hypersynchronous neuronal activity and metabolic demand^10^ and an initial reduction in tissue oxygenation occurs in normal brains, it is thus conceivable that NCSz might exceed the limits of NVC , potentially resulting in longer lasting brain tissue hypoxia.

A neural mass model (NMM) provides mathematical description of neuronal oscillation and EEG rhythms by modeling interactions of theoretical populations of different neurons. A NMM based on four neuronal population subgroups (main population of pyramidal cells, excitatory interneurons, slow inhibitory and fast inhibitory interneurons) showed that fast-spiking interneurons were crucial in inducing gamma oscillations^11^, which are one of the most characteristic intracranial EEG pattern in epileptic seizures. Studies have reported conflicting results on the modifications of cortical excitability during sepsis^12,13^.Thus, sepsis-induced modifications of meaningful parameters of this NMM might provide additional information on changes in cortical activity and on the neuronal component of the neurovascular unit.

In this study, we therefore aimed to assess whether NCSz lead to cerebral hypoxia in an ovine model of sepsis due to an abnormal NVC. Since sepsis is a gradual process with variable severity of organ dysfunction^14^, we also sought to compare the effects of NCSz during both isolated hyperdynamic phase without cardiovascular impairment and shock. We hypothesized that NVC would be more altered during shock and this might be associated with a more deleterious effect of NCSz on tissue oxygenation. We also investigated the sepsis-induced modification of different populations of modeled neurons in the NMM to clarify how sepsis modulates cortical activity. We finally evaluated the systemic effect of NCSz and the sepsis-induced alteration in cortical excitability in this setting.

We employed a well-controlled ovine model of sepsis that has already been used in previous studies on cerebral circulation and seizures^15,16^. Fecal peritonitis is characterized by a polymicrobial infectious focus that provides a scenario that is more similar to human sepsis, in term of clinical phases^17^ and cytokine profiles^18^, than systemic application of pro-inflammatory bacterial products (i.e. lipopolysaccharide).

## Results

### Parameters at time of seizure induction and seizure induction

Seventeen female sheep were included in the final analysis (6 in the sham, 5 in the sepsis and 6 in the septic shock group); one animal was excluded from the sepsis group, as it was found to be already septic at the time of delivery. A summary of systemic, biological parameters and information at the time of penicillin application is presented in Table 1. Except for MAP, which was lower in the septic shock group, groups did not show any significant difference concerning variables that could affect CBFv estimation or neuronal excitability, such as hemoglobin concentration, temperature, pH, PaO_2_ or PaCO_2_. Before correction for false discovery rate, pH was lower in the septic shock group than in the two others (*P* = 0.043 with sham and *P* = 0.07 with the septic group). The fast/delta ratio calculated before penicillin application was statistically lower in the septic shock group than the sham and sepsis groups, but did not differ between the sham and sepsis groups (Table 1).

**Table 1:** Summary of systemic, biological parameters and information at time of penicillin application in the studied animals.

Parameter	Sham	Sepsis	Septic shock	p values
	Median [IQR]	Median [IQR]	Median [IQR]	
CI_std (L/min/m^2^)	1.2 [0.9–1.3]	1.6 [1.5–1.9]	1.5 [1.3–1.6]	0.07
MAP (mmHg)	91^a^ [83–105]	77^a^ [77–90]	62 [53–64]	**0.02**
HR (bpm)	102 [96–109]	130 [130–153]	146 [133–152]	0.07
MPAP (mmHg)	12.6 [9–13]	7.7 [7–9]	11 [11–21]	0.08
PaCO_2_ (mmHg)	34.5 [33–35]	35.6 [33–35]	35.8 [34.8–39.1]	0.69
PaO_2_ (mmHg)	118 [117–120]	110 [108–114]	90 [74–107]	0.07
pH	7.44 [7.40–7.46]	7.44 [7.39–7.47]	7.31 [7.28–7.36]	0.07
Lactate (mmol/l)	0 [0–0]	1 [1–1.6]	2 [1.75–2.74]	0.07
Hb (g/dl)	8.3 [8.2–9.3]	9.4 [8.5–10.0]	8.8 [8.4–9.5]	0.64
T (°C)	40.0 [39.0–41.0]	39.5 [39.3–40.1]	40.6 [40.3–40.7]	0.24
PaO2/FiO2	385 [337–399]	378 [327–383]	191 [102–240]	0.07
Weight (kg)	29.5 [26.8–30]	29.5 [9, 26–32]	24.5 [24–25.8]	0.24
Latency to penicillin application (h)	6.2 [6.0–12.0]	8.5 [8.1–9.0]	12.6 [11.2–13.7]	0.14
Total penicillin dose (UI) × 10^4^	115 [85–145]	100 [100–110]	85 [50–97.5]	0.24
Latency to the 1st seizure (min)	118 [75–142]	81 [53–131]	NA [NA-NA]	0.62
Number of induced seizures	50 [9, 27–76]	37 [31–46]	NA [NA-NA]	0.78
Period of observation (h)	6.0 [5.5–7.0]	6.3 [4.7–9.7]	1.8 [1.3–2.8]	0.07
Cumulative MDZ dose (mg/kg)	7.3 [6.7–8.1]	10.3 [9.9–10.4]	8.7 [8.2–11.9]	0.64
Cumulative KET dose (mg/kg)	48.7 [45.0–54.3]	68.6 [65.7–69.5]	57.7 [54.5–79.2]	0.64
Fast/delta ratio	2.3^a^ [1.6–3.4]	2.3^a^ [1.3–2.9]	1.3 [1.1–2]	** < 0.01**

Data are presented as median and (IQR). CI_std: cardiac index standardized, expressed as variation of the cardiac index of the beginning of the experiment; MAP: mean arterial pressure; HR: heart rate; MPAP, mean pulmonary arterial pressure; Hb: hemoglobin; T °C: temperature in Celsius degrees; PaO_2_/FiO_2_: ratio used to identify and quantify acute lung injury (acute lung injury if ratio ≤ 300, acute respiratory distress syndrome if ratio ≤ 200). MDZ: midazolam; KET: ketamine; NA, not applicable. Fast-delta ratio of the ECOG signal was calculated before penicillin application. Variables were analyzed using the Kruskal–Wallis test. P-values were corrected for false discovery rate; the Dunn’s post hoc tests are presented in case of q-values < 0.05 (in bold in the table). ^a^significant at 5% vs. septic shock.

We observed no difference in the latency to the first seizure nor in the total number of seizures per animal between the sham and early sepsis groups (*P* = 0.88 and *P* = 0.83 respectively). Although the total dose of applied penicillin did not differ between groups, we were not able to induce seizures in the septic shock group. Of note, the total observation time was shorter, albeit non-significantly, in the septic shock group, since spontaneous death occurred. On the other hand, there were no differences between the total observed period in the septic shock group and the latency to the first seizure in other animals (*P* = 0.57).

### Cerebral parameters variations during seizures

**Figure 1:**
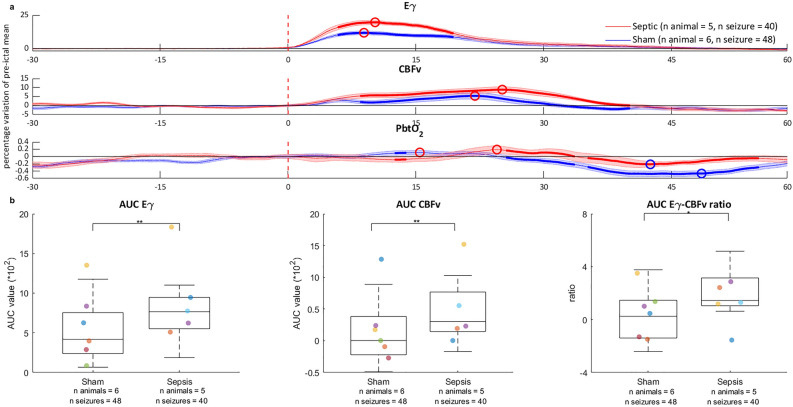
Local effects of seizures in the sham and sepsis groups (panel **a**) and comparison between groups of the area under the curve of the Eγ and of the CBFv variations and their ratio (panel **b**). Eγ: EEG gamma power envelope, CBFv: cerebral blood flow velocity, PbtO_2_: partial tissue oxygen pressure, AUC: area under the curve, *P < 0.01, **P < 0.001. Panel (**a**): in blue, mean ± SD seizure-induced value variation in time in sham group; in red, mean ± SD seizure-induced value variation in time in sepsis group. Mean and SD are calculated using bootstrapping with replacement and 100 iterations. Circles correspond to the peaks of a parameter variation (red circles for the positive and blue for the negative ones). Time 0 corresponds to the seizure onset (vertical dashed red line); curves were centered to zero at seizure onset. Differences between groups, identified by Wilcoxon Mann-Whitney rank sum test, are bolded and calculated on the raw data without bootstrapping. The increase in both CBFv and PbtO_2_ is delayed in the sepsis group (see text for “Discussion” section). Parameters are expressed in percentage of variation from the mean of the last 15 pre-ictal seconds except for Eγ, where baseline values were simply set to zero. Panel (**b**): boxplots of the AUC of the Eγ and of the CBFv variations and their ratio. An increase in the Eγ and the CBFv AUC was noted in the sepsis group (P < 0.001 for both).

**Figure 2:**
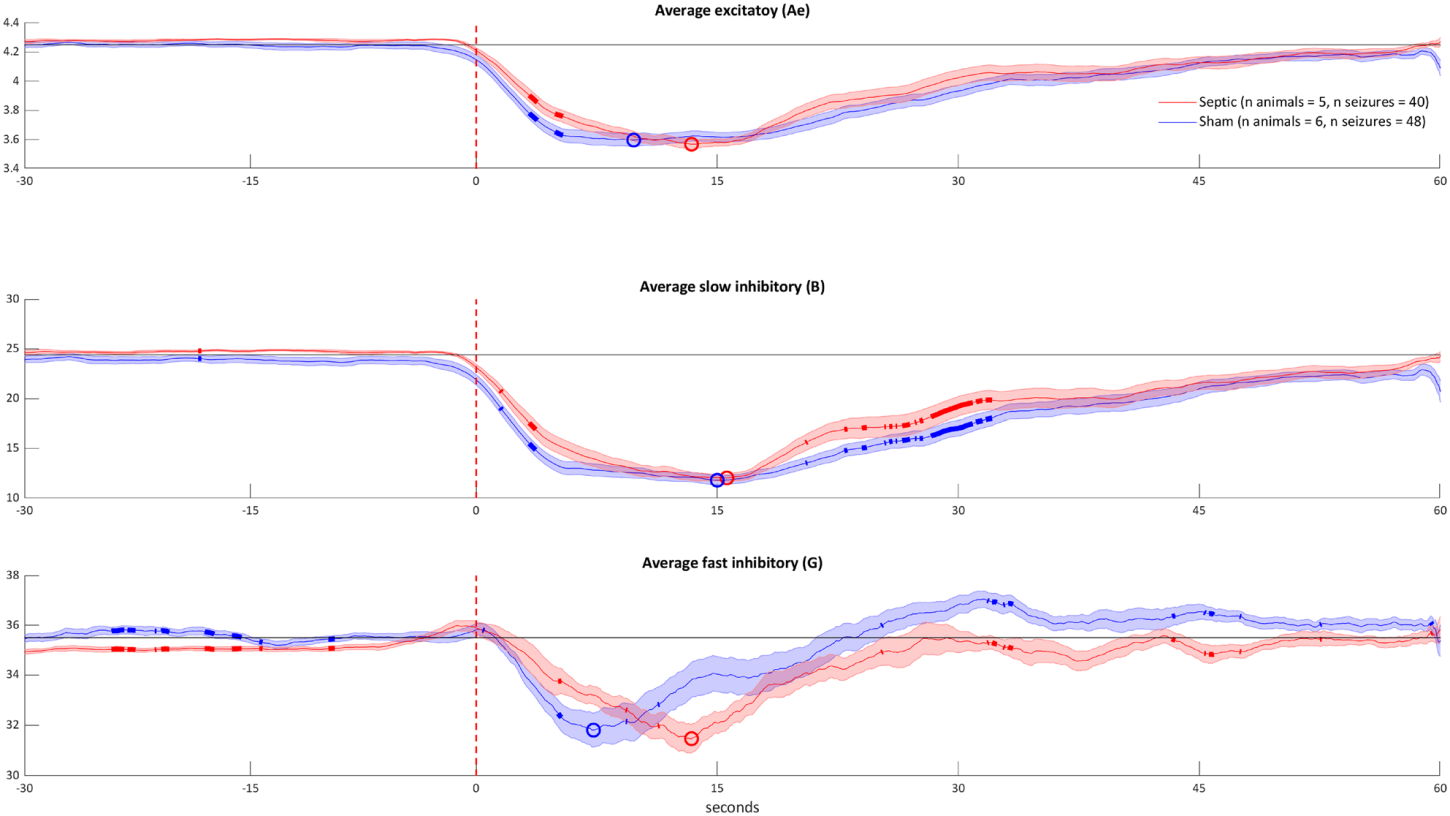
Temporal evolution of synaptic gain. In blue, mean ± SD seizure-induced value variation in time in sham group; in red, mean ± SD seizure-induced value variation in time in sepsis group. Mean and SD are calculated using bootstrapping with replacement and 100 iterations. Circles correspond to the peaks of a parameter variation (blue circles for the sham and red for the sepsis group). Time 0 corresponds to the seizure onset (vertical dashed red line). Differences between groups, identified by Wilcoxon Mann–Whitney rank sum tests, are bolded and calculated on the raw data without bootstrapping. For all the parameters, the descending part of the seizure-induced variation curve was steeper in the sham group. The dip was delayed in the sepsis group and particularly for the fast inhibitory synaptic gain variation that reached the dip with a lag of about 4.3 s (IQR [− 6.4–15] sec, *P* = 0.06, Table S3).

Figure 1, panel a shows the ictal variations of local and systemic parameters. In the sepsis group, the increase in the Eγ was greater throughout all the duration of the seizures than in the sham group. On the other hand, the ictal increase in the cerebrovascular parameters did not differ between the two groups, albeit the CBFv variation lasted longer in the septic animals. In the sepsis group, the AUC of ictal variations of both Eγ and CBFv was greater than in the sham group (P < 0.01, for both, Fig. 1, panel b). The variations in Eγ and CBFv were positively correlated in both groups (Spearman’s coefficient ρ = 0.4, *P* = 0.02 for shams & ρ = 0.44, *P* = 0.02 for sepsis, Supplementary material, Table S1). The Eγ /CBFv ratio was greater in the sepsis group (*P* = 0.002, Fig. 1, panel b). The positive ictal variation of the PbtO_2_ did not differ between groups (*P* = 0.66) but the post-ictal decrease in PbtO_2_ was both longer and deeper in the sham than in the sepsis group (*P* = 0.02). There were no statistically significant differences in the peak lags between groups (Supplementary material, Table S2). The temporal evolution of synaptic gains is shown in Fig. 2. We considered three meaningful parameters—the average excitatory (Ae), the slow and fast inhibitory synaptic gains (B and G) – which describe the interactions between four modeled populations of neurons (main population of pyramidal cells, excitatory interneurons, slow inhibitory and fast inhibitory interneurons). All the gains decreased during seizures in both groups but, in the sepsis group, a delayed onset of the variation in all the considered parameters was observed. Accordingly, the lag to the peak of the negative variation tended to be delayed, albeit not significantly (*P* = 0.67 for Ae, and *P* = 0.19 for G, Supplementary material, Table S3). The AUC of the synaptic gains tended to be lower in sepsis animals (Supplementary material, Fig. S4 and Table S3), but only for the fast inhibitory synaptic gain, G, the difference was significant (*P* = 0.001).

### Systemic hemodynamic parameters during seizures

**Figure 3:**
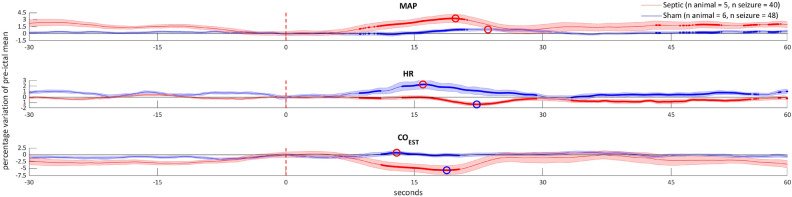
Systemic effects of seizures in the sham and sepsis groups. MAP: mean arterial pressure, HR: heart rate, CO_EST_: cardiac output estimation. See Fig. 1 for methodological explanations. The ictal MAP variation in the sepsis group is greater, whereas HR and CO_EST_ response in inverted in comparison to the sham group (see text for discussion).

In the sham group, after seizure onset, the increase in MAP was accompanied by a comparable increase in the HR and a slight rise in the CO (Fig. 3). The CO_EST_ and HR changes in the sepsis group showed an opposite pattern, since MAP increase coincided with a reduction of the CO_EST_ and of the HR. Furthermore, ictal MAP variation was greater in the sepsis group (8% [− 6.3 – 10.4] vs 2.5% [− 2.6 – 3.8]; *P* =  < 0.01).

## Discussion

In this work, we show that sepsis influences both cerebral and systemic responses to NCSz. The enhanced sepsis-induced EEG power during seizure is accompanied by a reduced NVC response.

The microcirculatory changes induced by sepsis, such as the reduction in the proportion of cortical perfused vessels^19^, the astrocyte end-feet^20^ and pericytes^21^ detachment from the vessel walls and increased the blood–brain-barrier permeability^22^ could alter the extracellular milieu of the brain, interfering with the neurovascular unit. Moreover, release of cytokines with a high vasoconstriction power, such as endothelin-1^23^, might move the physiological balance towards arteriolar vasoconstriction, either directly or by a reduction in endothelial nitric oxide production^24^. Consistent with these hypotheses, we showed that the seizure-induced cerebral vascular response in the sepsis group is relatively flattened. In fact, despite a more important neuronal and vascular activation, the Eγ/CBFv ratio was greater in the sepsis group, suggesting that the increase in neuronal activity was associated with a proportionally smaller CBFv variation than in shams. Possibly because of this reduced vascular response to neuronal activation, the rise in the PbtO_2_ tended to be delayed. These findings agree with previous studies that suggested that sepsis alters but not completely abolishes the NVC^25,26^. Indeed, we showed a persistent positive correlation between neuronal activation and vascular response in both groups. As sepsis progresses, more severe impairment of NVC, as previously demonstrated^9^ could be partially responsible for the alteration of brain function, which might prevent seizure occurrence, as observed in this study.

Interestingly, the altered NVC does not translate into an immediate neuronal dysfunction or substantial tissue hypoxia. Although mitochondrial dysfunction has been shown to occur in sepsis^27^, auxiliary sources of energy than mitochondrial oxidative respiration, such lactate or ketone bodies, might thus support neuronal activity^28,29^ in case of seizures. In line with this hypothesis, the physiological PbtO_2_ dip after seizure offset^30^ was relatively smaller in sepsis animals, suggesting a reduced O_2_ consumption. Nevertheless, since we did not benefit from microdialysis data, this remains a conjecture. Since we did not found a reduction in cortical activity in sepsis animals (Table 1), we deemed less likely that the smaller changes in the PbtO_2_ would be due to a reduced cortical metabolic demand, as it has been shown to occur in sepsis^12^. As sepsis progresses, this cerebrovascular inefficiency could be partially responsible for the alteration of brain function, which might prevent seizure occurrence.

Animal studies have shown that systemic inflammation enhances cerebral excitability, as revealed by an increase in seizure susceptibility^31^. Innate immune cells (i.e. microglia) could play a major role in this pathological response^32^ and previous studies have demonstrated microglial activation in sepsis^33–35^. Moreover, some human studies suggested that sepsis was a significant risk factor for NCSz^1^ and probably for NCSE^2^. In line with them, an increase of seizure susceptibility would have been expected in the sepsis groups; instead, although a prominent ictal Eγ power in sepsis animals, no seizures were observed in the septic shock group, despite a similar penicillin dose. In severe septic animals, brain function and perfusion could be altered to such an extent to reduce the ability of neurons to fire synchronously and thus prevent seizure occurrence, as suggested in a human study^36^. Using the fast/delta ratio as a surrogate for cortical neuronal activity^37^, we showed that septic shock animals presented a lower ratio than sham or sepsis group, suggesting an impairment of neuronal function. Since sedation did not differ between groups, the anti-epileptic properties of sedative drugs should not have influenced our results. Although pH was not statistically different between groups, septic shock animals were in metabolic acidosis. Cerebral acidosis is one of the supposed mechanism that might stop seizure^38^. Blood–brain barrier is impermeable to H + so that cerebral pH remains constant in the face of acute severe metabolic acidosis^39^. However, since BBB disruption occurs in sepsis^40^, it is conceivable that systemic acidosis might have influenced cerebral pH and contributed to the absence of seizure in the septic shock group. Brain dysfunction is a well-known complication of sepsis (i.e. sepsis-associated encephalopathy—SAE)^41^ and it has been shown that increasing severity of brain dysfunction was associated with progressing slowing of brain activity^42^, resulting in a decrease in the fast/delta ratio. The presence of a sepsis-induced NVC alteration, as we have shown with the reduced hemodynamic response to the increase in Eγ power in sepsis animals, might play a crucial role in SAE development. In fact, as the fast/delta ratio was not statistically different between sham and sepsis animals, our data support the hypothesis that, in sepsis, NVC impairment precedes neuronal dysfunction^6^, which successively develops when septic shock occurs^9^. Since no seizure occurred in septic shock animals, we were not able to test the NVC in this scenario and in particular to answer the question of whether seizures are associated with a reduction in tissue oxygenation during septic shock.

We used the NMM to estimate if a difference in temporal evolution of the synaptic activity of the modeled neuronal populations might explain the differences in neuronal activation and in the cerebrovascular hemodynamic response in septic animals. It has been shown that sepsis induces variations in important neurotransmitter metabolisms, such as those of acetylcholine^43^, noradrenaline^44^ and gamma-aminobutyric acid (GABA)^45^, and this might play a role in SAE. For all the analyzed parameters, we noticed a deflection during seizure. These alterations are consistent with what was previously reported in the literature^46^ and they suggest an excitation/inhibition imbalance leading to seizure. Of note, the variations were delayed and blunted in the sepsis group, suggesting a certain extent of synaptic impairment induced by sepsis which might not be homogenous between neuronal populations as it happens for ischemic insults^47^. Therefore, the activity of the modeled fast somatic-projecting inhibitory interneurons, which are described by the fast inhibitory synaptic gain G in the NMM model, was less reduced in the sepsis group. Since these interneurons might play a role in inducing gamma oscillations^11^, this finding may partially explain the more prominent Eγ power in sepsis. Furthermore, we may speculate that the delayed activation of neural populations in sepsis might explain the delayed seizure-induced peak in CBFv increase we recorded. Finally, this excitation/inhibition imbalance might furtherly worst as sepsis progress, contributing to the negative influence of an altered NVC in preventing seizure occurrence in septic shock. Of note, since the considered neuronal populations in the NMM are theoretical and the model simplifies an extremely complex twine of neuronal interactions, this part of our conclusion remains speculative.

The ictal systemic hemodynamic response differences between groups are intriguing but hard to explain. Although cardiovascular effects of NCSz are a matter of debate, sympathetic outflow seems to be enhanced during seizures^48^. The systemic cardiovascular response in the sham group reflects the physiologic effect of sympathetic stimulation leading to a slight increase in MAP accompanied by a chrono- and inotropic cardiac effect^49^. In the sepsis group, an increased α adrenergic receptor (AR) activity^50^ might mediate a rise in MAP by systemic vasoconstriction, inducing a baroreceptor reflex activation and, thus, a reduction in HR and CO_EST_. Moreover, an adrenal insufficiency, which has been reported in sepsis^51^, may reduce the positive cardiac adrenergic effect of adrenaline; the rise in BP would no longer be combined to a positive chrono- et inotropic effect but by a negative one mediated by baroreceptor reflex. Data interpretation might be furtherly complicated by the interaction of other mechanisms influencing cardiovascular physiology, such as arginine-vasopressin (AVP) and the renin-angiotensin systems (RAS). It has been shown that they share superimposable inotropic and peripheral-vasoconstriction effects in healthy subjects^52,53^. Therefore, despite an increase of AVP has been reported after convulsive seizures^54^, it is challenging to establish if the rise in plasma concentration is a direct effect of seizure or it is an epiphenomena of the physiologic anomalies accompanying convulsion (i.e. hypotension, hypoxia, autonomic nervous system activation^55^). Moreover, the role of these hormones in non-convulsive seizure is unknown. Several studies has shown that sepsis induces a depletion of plasmatic levels of AVP and a downregulation of angiotensin receptors in vascular bed^56,57^. It is thus conceivable that the sepsis-induced alteration in these physiologic systems might influence the cardiovascular response to seizure that we recorded in sepsis animals. Further research is needed to explore the ictal variations of AVP and RAS in non-convulsive seizures.

Previous studies have essentially focused on local cerebral mechanisms of CBF regulation, especially NVC, to describe the vascular response to seizure^7,58^. However, evidence from both humans and animals experiments suggests a close relationship between CO and CBF^59^ and a study in septic patients showed a linear relationship between cardiac index and cerebral blood flow^60^. Despite the difficulty to distinguish the net effects of cerebral and systemic factors influencing CBF, we may speculate that the alteration of systemic hemodynamic, recorded in sepsis animal, especially the reduction in the ictal CO_EST_, might negatively influence the physiologic CBF response to neuronal activation.

Our study presents several limitations. First, regarding the latency to seizure onset in septic shock group, one could speculate that, since a neuronal dysfunction occurs in severe sepsis, the time necessary to induce seizure in the septic shock group could have taken longer than in other groups. Since specific sepsis therapies, such as antibiotics, might enhance seizure excitability^61^, we preferred not to employ them; thus, this hypothesis could not be verified since septic shock animals spontaneously succumbed. However, human data seem to support our presumption that severe sepsis-induced cerebral dysfunction prevents seizure occurrence^36^. Secondly, the variations in tissue oxygenation we recorded are smaller than those reported in other animal studies^7,58^. Our data might be the average of the ictal focus and surrounding tissue PbtO_2_ variations, which are not identical and, in fact, often opposite. Furthermore, since we did not measure any metabolic parameters, we could not definitely conclude to a reduced oxygenation consumption. Of note, no specific intra-group variations or inter-groups differences in PbtO_2_ values were recorded during resting-state conditions. Thirdly, NMM privileges the description of key mechanisms of neuronal networks using simplified assumptions and empirical priors^62^, which has been successful in reproducing EEG signals in patients^63^. Thus, it is possible that the set of parameters fitting humans may not be appropriate for our experimental model. Fourth, since we did not perform power analysis to define sample size, our results await confirmation from more numerous cohorts. Fifth, the sepsis group received a cumulative dose of sedative drugs 40% higher than sham group, albeit not significantly (*P* = 0.14), because seizures were induced slightly later in these animals than in controls (8.5 vs 6.2 h, *P* = 0.14). Although it is possible that drug metabolism in the sepsis group may have been reduced by sepsis-induced multi-organ failure, it has to be noted that the number of seizures were not statistically different between groups (median 37 vs. 50, respectively, *P* = 0.83). Moreover, the amplitude of seizure-induced cortical response Eγ was statistically higher in the sepsis group (Fig. 1). Thus, there are no clear arguments to support the hypothesis that anesthesia significantly influenced our results by reducing cortical excitability in the sepsis group. It has been shown that ketamine might increase regional and global CBF in both humans and animals studies^64^. Since we calculated the percentage of seizure-induced CBF variation from the baseline, the absolute value of initial CBF should not have influenced our findings. Finally, the influence of cerebral autoregulation (CA), defined as the capacity of the brain to maintain an adequate cerebral blood flow despite variations in cerebral perfusion pressure^65^, could be considered as a confounding factor in the interpretation of the link between systemic and local variables. As sepsis and seizures seem to alter CA^66,67^, CBFv variations could be influenced by MAP fluctuations rather than by the sepsis-induced NVC alteration. Moreover, since we did not find significant differences in the correlation between EEG/CBFv ratios and ictal-induced CO_EST_ variations between groups, the negative influence of the reduction in the ictal CO_EST_ on the CBF response to neuronal activation remains a matter of speculation.

## Conclusions

In conclusion, our study shows that sepsis alters both local and systemic responses to seizures. The NVC is blunted in sepsis, contributing to the alteration in brain function that develops as sepsis progress, and possibly preventing seizure occurrence in septic shock. The alteration of the systemic hemodynamic support to cerebral perfusion might affect cerebrovascular response to neuronal activation. These anomalies probably contribute to the sepsis-associated encephalopathy.

## Methods

### General procedure

Animal model procedures have been previously described^9,16^. Briefly, the Institutional Review Board for Animal Care of the Free University of Brussels (Belgium) approved all experimental procedures (number of Ethical Committee approval: 675 N), which were also in compliance with ARRIVE (Animal Research: Reporting in Vivo Experiments) guidelines. Care and handling of the animals were in accord with National Institutes of Health guidelines (Institute of Laboratory Animal Resources). The protocol was performed on 18 female *Ovis Aries* sheep. Animals were allocated with a 1:1:1 ratio to a sham procedure (n = 6), early sepsis assessment (n = 6) and septic shock assessment (n = 6) groups. Sample sizes were based on previous studies from our laboratory using the same animal model^16^. After randomization, animals were excluded if they presented a hemoglobin level below 8 g/dL or systemic signs of infection at time of delivery to the laboratory. Left-side craniotomy was performed and dura mater opened to insert a 4-contact electrocorticography (ECOG) electrode (Dixi Medical, Besançon, France) over the sensory cortex surface. Near the ECOG electrode, the dura mater was subsequently punctured to insert a laser-Doppler flowmetry probe (OxyFlow 4000, Oxford Optronic, UK) for local cerebral blood flow velocity (CBFv) measurement and a Clark electrode (Licox; Integra Lifesciences, Zaventem, Belgium) for brain tissue oxygen partial pressure (PbtO_2_) measurement. Except for sham animals, feces were collected from the caecum through a midline laparotomy (1.5 g.kg−1 of body weight). A plastic tube was inserted through the laparotomy incision in the abdominal cavity for successive feces injection.

### Monitoring and measurements

Monitoring protocol has been previously published^9,16^. Briefly, a continuous IV infusion of ketamine, morphine, and midazolam was used as general anesthesia throughout the entire experiment adjusting initial doses to achieve a nearly continuous EEG background^68^. Ventilator parameters were adjusted to maintain PaO_2_ and PaCO_2_ values in the normal ranges. Mean arterial pressure (SC 9000 monitor; Siemens, Berlin, Germany), heart rate (HR), core-temperature and cardiac output (CO) (Vigilance II monitor; Edwards Lifesciences, Irvine, California, United-States) were monitored continuously. Systemic hemodynamic parameters, ECOG, PbtO_2_, and CBFv were recorded continuously and simultaneously with a sampling rate of 250 Hz (Notocord-hem, Instern Company, France). Measurements of mean pulmonary arterial pressure were collected every 1.5 h. Cardiac index (CI) was calculated using standard formulas; the body surface area was estimated from Mitchell’s sheep-specific formula^69^.

### Experimental protocol

After surgical procedures, the animal was allowed to stabilize. In all septic animals, feces were injected into the abdominal cavity. Seizures were induced by topical application of penicillin G on the cerebral cortex beneath the left-sided ECOG electrode; 10^5^ penicillin UI, diluted in 0.1 mL of CNS perfusion fluid (Perfusion Fluid CNS, CMA Microdialysis AB, Sweden), were applied every 15 min until seizures were induced (Supplementary material, Fig. S1). Of note, since animals were paralyzed, seizures were all non-convulsive. In the sepsis group, penicillin application occurred during the hyperdynamic phase of sepsis, arbitrarily defined when the cardiac index (CI) exceeded by 130% and the pulse pressure (PP) by 140% their baseline values. At the end of the experiment, animals were sacrificed with a bolus of IV potassium chloride. In the septic shock group, penicillin was applied after the onset of shock, defined, according to the last consensus definitions^14^, as MAP < 65 mmHg persisting more than 15 min and lactate elevation > 2 mmol/L despite adequate fluid resuscitation. Data were collected until spontaneous death occurred.

### Study outcomes

The primary outcome of the work was to study the effects of sepsis on NVC during non-convulsive seizures. Our hypothesis was that non-convulsive seizures, occurring during sepsis, might further impair NVC and result into brain tissue hypoxia. The second aims of the work were to assess systemic hemodynamic responses to seizures and to analyze the influence of sepsis on cortical excitability.

### Data analysis

Cerebral and systemic parameters were analyzed as previously described^16^. All analyses were performed off-line, using built-in and custom functions in Matlab (The MathWorks, Natick, MA, USA). Physiologically implausible measures and outliers, defined according to Chauvenet’s criteria^70^, were removed. Seizure onsets were defined as rhythmic discharges with a frequency ≥ 3 Hz, according to consensus criteria^68^. For each detected seizure, ECOG epochs from 30 s before to 60 s after seizure onset were selected (Supplementary material, Fig. S2); if the inter-ictal interval between two consecutive seizures was shorter than 60 s, both seizures were discarded. The envelope of the low gamma frequency band (30–50 Hz) (Eγ) was extracted from the ECOG signal using wavelet transform spectral density estimate (Morse wavelet; *cwt*, *icwt* and *envelope* functions in Matlab). Corresponding physiological variables (MAP, HR, CBFv, PbtO_2_ and CO) were considered. CBFv signal was high-pass filtered with a cut-off frequency of 0.25 Hz. Linear interpolation was used to replace excluded data. Finally, physiological variables were expressed as percentage variations from baseline using the mean calculated on the pre-ictal 15 s period, as previously described^71^, and their value set to 0 at seizure onset; for Eγ, baseline values were simply set to zero. Local (CBFv and PbtO_2_) and systemic (MAP, HR, CO_EST_) parameters were compared between groups, taking the first 8 analyzable seizures for each animal of each group, in order to have a substantial sample size for statistical analysis thus limiting the number of animals needed. For each parameter, the value of the peak of the ictal maximal absolute deviation from the pre-ictal baseline, its lag from the seizure onset and the area under the curve (AUC) of its variation were considered. To assess the NVC response, we compared the ratio of the AUC of the Eγ and that of the CBFv between groups.

We analyzed the evolution of EEG activity during seizures using a neural mass model (NMM), which was shown to accurately reproduce real EEG signals^63^. NMM provides mathematical description of neuronal oscillation and EEG rhythms by modeling average properties and interactions of a large numbers of neurons, i.e. cortical pyramidal cells and interneurons. Previous studies suggested that two classes of inhibitory interneurons, slow dendritic-projecting inhibitory interneurons and fast somatic-projecting inhibitory interneurons, play a role in formation of EEG rhythms^72^. In particular fast-spiking interneurons were crucial in inducing gamma oscillations^11^, which are one of the most characteristic intracranial EEG pattern in epileptic seizures. These evidences were successfully implemented in a NMM of epilepsy in humans^46,63^ based on four neuronal population subgroups (main population of pyramidal cells, excitatory interneurons, slow inhibitory and fast inhibitory interneurons). The NMM was automatically fitted to EEG to analyze the temporal evolution (between 30 s before to 60 s after seizure onset) of three meaningful parameters—the average excitatory (Ae), the slow and fast inhibitory synaptic gains (B and G) – as previously proposed^46,63,73,74^. We calculated the AUC and the lag from the beginning of the seizure to the peak of the variation for each parameter.

Spectrograms were calculated using the wavelet transform spectral density estimate (Morse wavelet; *cwt* function in Matlab). To represent the integrity of cortical neuronal activity, the fast-delta ratio (FDR) was further calculated, as the ratio between the sum of the power in the gamma (15–100 Hz) and beta (8–14 Hz) frequency bands divided for the power of the delta (0.1–4 Hz) ones^9^.

### Statistical analysis

Statistical analyses were performed using Matlab (MathWorks) and SPSS for Windows (IBM SPSS Statistics 25, Chicago, IL). A p value < 0.05 was considered statistically significant. Data are presented as mean ± SD or median and [IQR]. Kruskal–Wallis test was used to assess differences between groups. The linear step-up procedure introduced by Benjamini and Hochberg was applied for controlling the false discovery rate^75^. Dunn’s post-hoc analysis was employed in case of a q-value < 0.05. To compare ictal-induced local and systemic parameters changes between groups, point by point repeated Wilcoxon/Mann–Whitney rank sum tests were performed (*ranksum* function in Matlab).

The AUC, the peak amplitude and the lag of the ictal cerebral parameters variations from the baseline were also compared between groups using a Wilcoxon test. The Spearman’s rank-order correlation coefficient was used to measure the strength of the association of the ictal variations of Eγ and those of PbtO_2_ and CBFv. In fact, previous studies showed a positive linear correlation between epileptic neuronal activity and the amplitude and the duration of cerebrovascular parameters^76^. To compare the vascular response to neuronal activation of sham and sepsis groups, we used the ratio between the AUC of the Eγ and that of the CBFv, which was supposed to remain stable over time. Synaptic excitatory (Ae) and inhibitory (G and B) gain variations during seizures were compared, point by point, between the sham and sepsis groups with the corresponding gains in septic animals using a repeated Wilcoxon/Mann–Whitney rank sum tests (*ranksum* function in Matlab).

To ensure that all measured parameters were stable over time before seizure induction, 8 consecutives 90 s periods were selected within the 90 min before penicillin application and analyzed as reported above for seizures. Results are presented in Supplementary material, Fig. S3.

## Supplementary Information

Supplementary Information.

## Data Availability

The data underlying this article will be shared on reasonable request to the corresponding author.
