# Supplementary material for: Sepsis modulates cortical excitability and alters the local and systemic hemodynamic response to seizures
Source: Sci Rep. 2022 Jul 5;12:11336. doi: 10.1038/s41598-022-15426-w (PMC9256588; doi:10.1038/s41598-022-15426-w)
Supplement: Supplementary file 1 — Supplementary Information. [file 41598_2022_15426_MOESM1_ESM.docx]

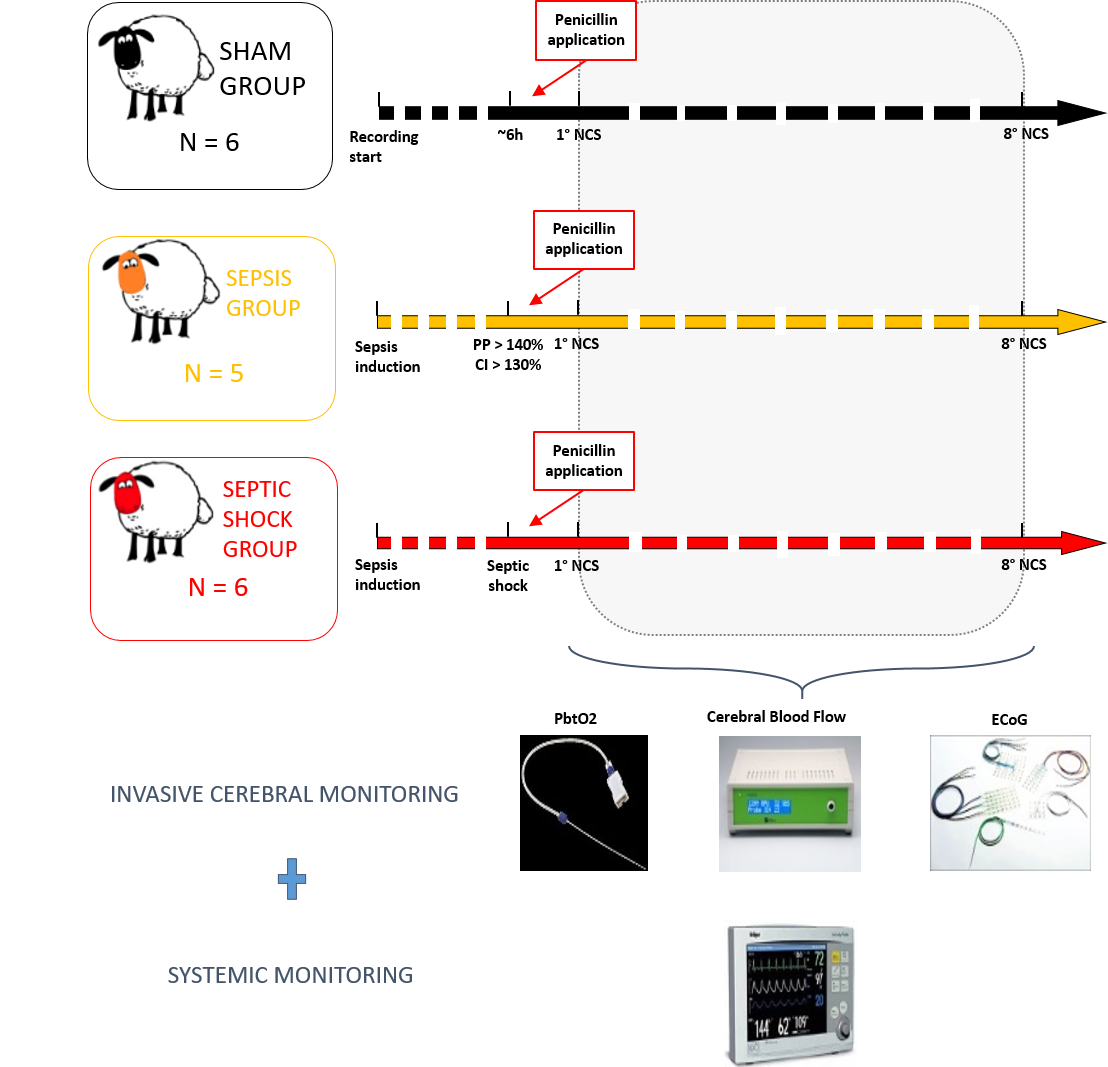
**SUPPLEMENTARY MATERIAL**

**Figure S1. Study protocol.** Abbreviation: NCS: nonconvulsive seizure, PP: pulse pressure, CI : cardiac index, PbtO_2_: partial tissue oxygen pressure, ECoG: electrocorticography electrodes. Cerebral (PbtO2, cerebral blood flow, neuronal activity) and systemic variables (mean arterial pressure, cardiac output, heart rate) between the first and the eighth induced seizures were recorded for further analysis (grey rectangle, see figure S2). In the sham group, seizure induction occurred after a median of 6.3 (IQR [6-12]) hours from surgical procedures. In the sepsis group, seizures were induced when the pulse pressure and the cardiac index overcome the threshold of 140 and 130% of their value after surgical procedures respectively, after 8.5 ([8.1-9]) hours from surgery (for more details, please refer to the text). In the septic shock group, seizures were induced when the serum lactate overcame 2 mmol/L and mean arterial pressure fell below 65 mmHg despite fluid resuscitation (12.6 [11.2-13.6] h after surgical procedures).


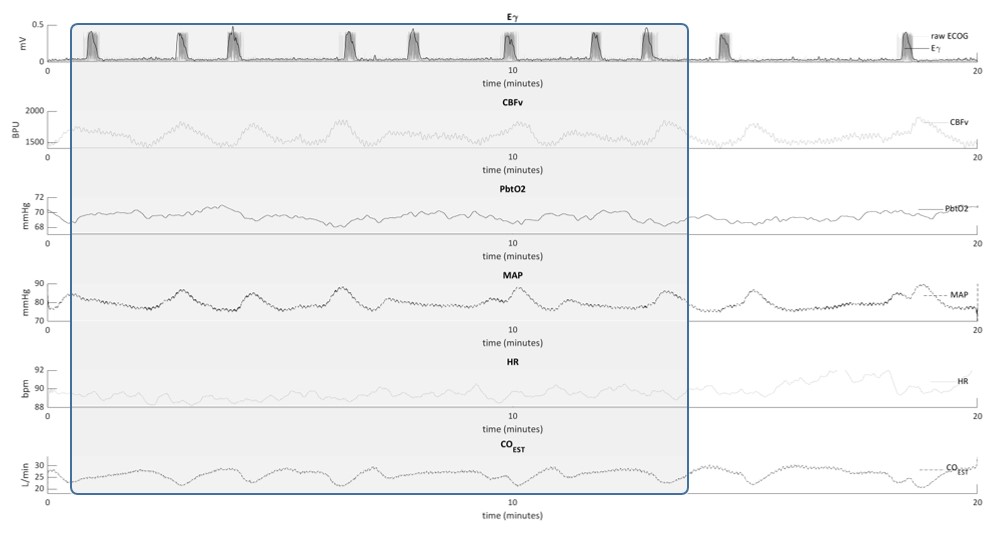
**Figure S2. Multimodal cerebral and systemic recording in an animal with penicillin-induced seizures.** Abbreviations: Eγ: EEG gamma power envelope, CBFv: cerebral blood flow velocity, PbtO_2_: partial tissue oxygen pressure, MAP: mean arterial pressure, HR: heart rate, CO_EST_: cardiac output estimation. Time = 0 corresponds to the last penicillin application before seizure occurrence. Peaks in the gamma envelope of the ECOG signal corresponds to induced seizures on electrocorticography (ECOG – top row). Seizures are associated with time-locked variations in local (cerebral blood flow velocity [CBFv –second row], partial brain tissue oxygen pressure [PbtO2 –third row) and systemic (mean arterial pressure [MAP] –forth row, heart rate [HR] – fifth row, and estimated cardiac output [COest] – last row) parameters. The first 8 considered seizures are embedded in the grey shape (for more details, please refer to the text).

**
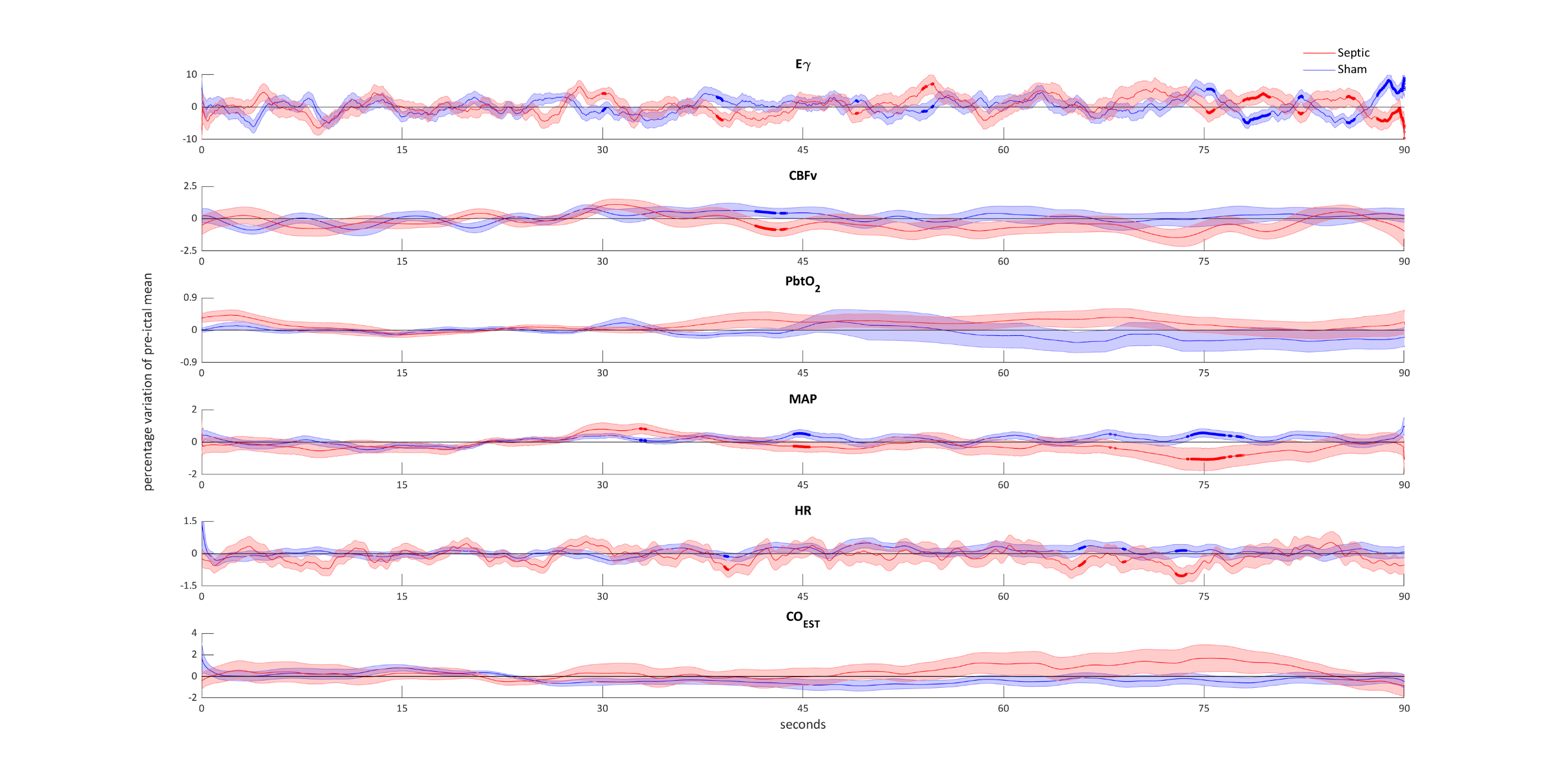
Figure S3. Local and systemic variations in resting state conditions.** Abbreviations: Eγ: EEG gamma power envelope, CBFv: cerebral blood flow velocity, PbtO_2_: partial tissue oxygen pressure, MAP: mean arterial pressure, HR: heart rate, CO_EST_: cardiac output estimation. In blue, mean ± SD seizure-induced value variation in time in sham group; in red, mean ± SD seizure-induced value variation in time in sepsis group. Mean and SD are calculated using bootstrapping with replacement and 100 iterations. Curves were centered to zero at seizure onset. Differences between groups, identified by Wilcoxon/Mann-Whitney tests, are bolded and calculated on the raw data without bootstrapping. Parameters are expressed in percentage of variation from the mean of the last 15 pre-ictal seconds. There are neither clear spontaneous variations of parameters nor
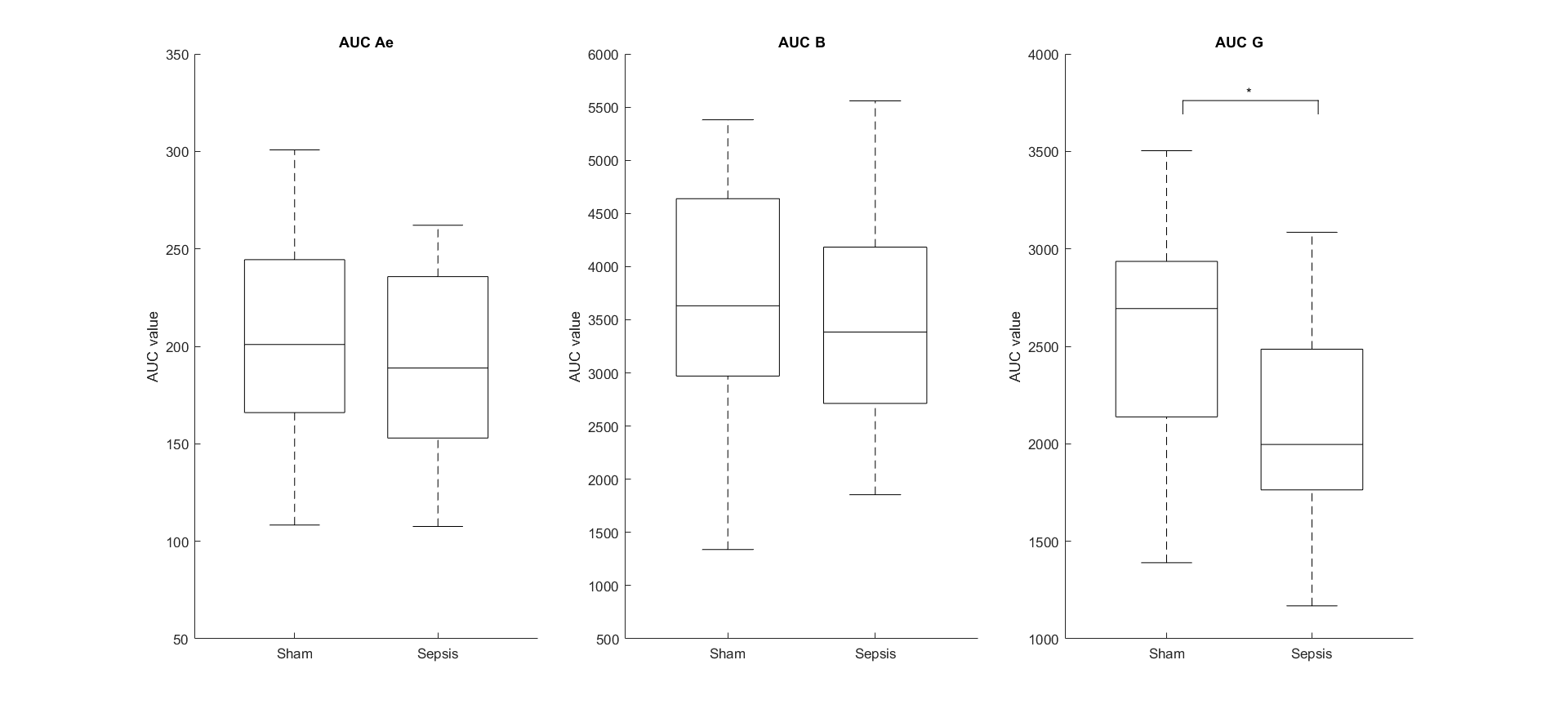
significant differences between groups.

**Figure S4. Comparison between groups of the area under the curve of the variations of the synaptic gain parameters.** Abbreviations: AUC : area under the curve, Ae : average excitatory synaptic gain, B: average slow inhibitory synaptic gain, G: average fast inhibitory synaptic gain. * : P < 0.01. The AUC of all parameters were higher in the sham group, but only for the fast inhibitory synaptic gain, G, the difference was significant (P = 0.001, cf. **Table S2**).

**Table S1. Correlations between the AUCs of the ictal variations of cerebral parameters.**

|  | SHAM | | SEPSIS | |
| --- | --- | --- | --- | --- |
| AUC correlation | Spearman’s coefficient | q | Spearman’s coefficient | q |
| Eγ - CBFv | 0,40 | **0,02** | 0,44 | **0,02** |
| Eγ - PbtO2 | 0,08 | 0,57 | 0,01 | 0,97 |
| Eγ - PbtO2 dip | 0,18 | 0,28 | -0,01 | 0,97 |
| CBFv - PbtO2 | -0,29 | 0,09 | 0,21 | 0,37 |
|  |  |  |  |  |

Abbreviations: AUC: area under the curve, Eγ: EEG gamma power envelope, CBFv: cerebral blood flow velocity, PbtO2: partial tissue oxygen pressure. P-values were corrected for false discovery rate (q-values); q-values < 0.05 are in bold in the table.

**Table S2. Comparisons between the amplitude and the lag of the peaks of the ictal variations of cerebral and systemic parameters between groups.**

| Parameter | | Peak variation (median [IQR] %) | | | | | | q | Latency of the peak (median [IQR] sec) | | | | | | q |
| --- | --- | --- | --- | --- | --- | --- | --- | --- | --- | --- | --- | --- | --- | --- | --- |
| **Eγ** | Sham | 9.0 | [ | 3.9 | - | 14.8 | ] | **<0.001** | 7.7 | [ | 4.8 | - | 11.1 | ] | 0.389 |
|  | Septic | 18.5 | [ | 16.8 | - | 25.9 | ] |  | 7.7 | [ | 5.5 | - | 13.0 | ] |  |
| **CBFv** | Sham | 7.2 | [ | 3.7 | - | 16.3 | ] | 0.290 | 21.6 | [ | 14.8 | - | 29.8 | ] | 0.795 |
|  | Septic | 8.8 | [ | 6.1 | - | 15.0 | ] |  | 22.0 | [ | 15.3 | - | 28.8 | ] |  |
| **PbtO_2_** | Sham | 0.1 | [ | -0.3 | - | 0.5 | ] | 0.832 | 15.5 | [ | 11.6 | - | 20.2 | ] | 0.255 |
|  | Septic | 0.1 | [ | -0.5 | - | 0.5 | ] |  | 17.3 | [ | 12.9 | - | 21.2 | ] |  |
| **MAP** | Sham | 2.6 | [ | -2.7 | - | 3.9 | ] | **<0.001** | 14.0 | [ | 7.4 | - | 27.9 | ] | 0.232 |
|  | Septic | 8.1 | [ | -6.4 | - | 10.5 | ] |  | 25.2 | [ | 8.6 | - | 31.1 | ] |  |
| **HR** | Sham | 2.1 | [ | -1.3 | - | 3.4 | ] | **<0.001** | 22.7 | [ | 13.4 | - | 32.4 | ] | 0.325 |
|  | Septic | -2.5 | [ | -3.4 | - | 2.4 | ] |  | 26.5 | [ | 19.1 | - | 32.0 | ] |  |
| **CO_EST_** | Sham | 1.0 | [ | -5.4 | - | 5.7 | ] | **0.003** | 16.8 | [ | 8.3 | - | 26.5 | ] | **0.007** |
|  | Septic | -11.8 | [ | -24.0 | - | 9.1 | ] |  | 26.9 | [ | 12.9 | - | 35.7 | ] |  |

Abbreviations: Eγ: EEG gamma power envelope, CBFv: cerebral blood flow velocity, PbtO2: partial tissue oxygen pressure, MAP: mean arterial pressure, HR: heart rate, CO_EST_: cardiac output estimation. P-values were corrected for false discovery rate (q-values); q-values < 0.05 are in bold in the table.

**Table S3. Comparisons between the AUC and the lag of the ictal variations of synaptic gain parameters between groups.**

| Parameter | | SHAM | | SEPSIS | | p value |
| --- | --- | --- | --- | --- | --- | --- |
|  |  | Median | IQR | Median | IQR |  |
| AUC | Ae | 201 | [166 - 244] | 189 | [153-236] | 0.38 |
|  | B | 3631 | [2970-4639] | 3384 | [2713-4183] | 0.32 |
|  | G | 2694 | [2138-2936] | 1997 | [1764-2485] | **< 0.001** |
| LAG (sec) | Ae | 17.9 | [10.1-30.1] | 18.1 | [11.3-31.3] | 0.55 |
|  | B | 16.8 | [9.1-25.5] | 16.4 | [11.2-28.6] | 0.84 |
|  | G | 16.3 | [10.7-26.3] | 22.6 | [15.6-32.3] | 0.06 |

Abbreviations: AUC: area under the curve, Ae : average excitatory synaptic gain, B: average slow inhibitory synaptic gain, G: average fast inhibitory synaptic gain. Variables were analyzed using the Wilcoxon-Mann-Whitney test and P-values; significant p-values at 5% are bold. There is a significant difference in the AUC of the variation in the G parameter, indicating that the reduction in average fast inhibitory synaptic gain in sepsis was lower than in sham.
